# Endoscopic third ventriculostomy and ventriculoperitoneal shunt for patients with noncommunicating hydrocephalus: A PRISMA-compliant meta-analysis

**DOI:** 10.1097/MD.0000000000012139

**Published:** 2018-10-19

**Authors:** Lin Jiang, Guangzhong Gao, Yanfeng Zhou

**Affiliations:** Department of Neurosurgery, Taizhou People's Hospital, Taizhou, Jiangsu Province, China.

**Keywords:** endoscopic third ventriculostomy, hydrocephalus, ventriculoperitoneal shunt

## Abstract

**Background:**

The surgical methods of endoscopic third ventriculostomy (ETV) and ventriculoperitoneal shunt (VS) for patients with noncommunicating hydrocephalus have rapidly increased in the past 2 decades. However, there is controversy regarding the efficacy and safety of these 2 surgical methods for noncommunicating hydrocephalus. The purpose of this study was to identify whether ETV is safer and more efficacious than VS for patients with noncommunicating hydrocephalus.

**Methods:**

We performed electronic searches in PubMed, Embase, China National Knowledge Internet, and the Cochrane Library to identify studies published up to February 03, 2018. The study summary results included improvement of symptoms, major complications, hematoma, infection, reoperation, mortality, duration of surgery, and hospital stay. Odds ratios (ORs) or standard mean differences (SMDs) with 95% confidence intervals (CIs) were calculated using random-effects models.

**Results:**

We identified 10 observational studies (4 prospective and 6 retrospective studies) with data collected from 2017 patients with noncommunicating hydrocephalus. First, there was no significant difference between ETV and VS for symptom improvement (OR: 0.83; 95%CI: 0.46–1.50; *P* = .534). Second, ETV was associated with lower incidence of major complications when compared with VS (OR: 0.31; 95%CI: 0.17–0.56; *P* < .001). Third, ETV has little or no significant effect on hematoma (OR: 0.65; 95%CI: 0.22–1.92; *P* = .433) and mortality (OR: 0.90; 95%CI: 0.11–7.72; *P* = .926). Fourth, ETV were associated with lower incidence of infection (OR: 0.20; 95%CI: 0.06–0.69; *P* = .010) and reoperation (OR: 0.22; 95%CI: 0.08–0.56; *P* = .002). Finally, patients who received ETV had shorter duration of surgery (SMD: -1.71; 95%CI: -3.16 to -0.27; *P* = .020) and hospital stay (SMD: −0.91; 95%CI: −1.45 to −0.38; *P* = .001).

**Conclusions:**

This meta-analysis provides robust evidence that ETV has greater benefits in terms of major complications, infection, reoperation, duration of surgery, and hospital stay than VS for patients with noncommunicating hydrocephalus.

## 1 Introduction

Hydrocephalus is one of the commonest complications of tuberculous meningitis, including noncommunicating, communicating, and combinations of obstruction in addition to defective absorption of cerebrospinal fluid.^[1–3]^ The pathophysiology of hydrocephalus is complicated and remains unclear, which creates challenges in the management of patients with hydrocephalus. Currently, endoscopic third ventriculostomy (ETV) and improved shunt hardware are widely used for patients with noncommunicating hydrocephalus, while controversy persists regarding optimal treatment.^[4–10]^

Placement of a shunt as a standard treatment strategy has been in use for numerous years, while the incidence of shunt failure has remained similar to that from 40 years ago.^[11–14]^ Further, the use of advanced neuroimaging systems is associated with earlier diagnosis and has led to the combined use of corticosteroids, direct nidus removal, and external ventricular drainage.^[15–18]^ Therefore, ETV is employed as a renascence for the treatment of noncommunicating hydrocephalus. Although both techniques are effective in treating hydrocephalus, there seems to be lack of evidence supporting the rapid evolution of the endoscopic technique and surgeons are usually expected to rely on their experience.

A previous meta-analysis found a similar therapeutic effect between ETV and ventriculoperitoneal shunt (VS) for patients with noncommunicating hydrocephalus, while ETV was associated with lower incidence of major complications, reoperation, and duration of surgery.^[19]^ However, the treatment effects in patients with specific characteristics have not been elucidated. Another important meta-analysis found that both ETV and VS were associated with higher failure rates, with no significant difference between the 2 techniques, while numerous other indexes, including adverse events and variability in surgery were not evaluated.^[20]^ Clarifying the optimal techniques for noncommunicating hydrocephalus is particularly important and has not been definitively determined. Here, we attempted a comprehensive examination of available studies to determine the best treatment strategy for noncommunicating hydrocephalus and evaluated the treatment effects of ETV and VS in specific subsets.

## 2 Methods

### 2.1 Data sources, search strategy, and selection criteria

This review was conducted and reported according to the Preferred Reporting Items for Systematic Reviews and Meta-Analysis Statement issued in 2009 (Checklist S1).^[21]^ Ethics approval was not necessary for this study, as only de-identified pooled data from individual studies were analyzed. A comprehensive search was performed for potentially suitable studies published in English or Chinese in the electronic databases of PubMed, Embase, China National Knowledge Internet, and Cochrane library up to February 03, 2018. The core keywords included (“endoscopic” OR “ETV”) AND (“shunt” OR “VS”) AND “third ventriculostomy” AND “hydrocephalus” AND “human”. We also conducted manual searches of reference lists from all the relevant original and review articles to identify additional eligible studies. The study title, study design, disease status, exposure, control, and outcome variables of identified articles were considered to select the relevant studies.

Two authors searched for articles and reviewed of all retrieved studies independently using a standardized approach. Disagreements between the 2 investigators were resolved by group discussion until a consensus was reached. All studies comparing the efficacy and safety of ETV and VS for patients with noncommunicating hydrocephalus were considered for inclusion. The inclusion criteria were as follows: studies with prospective or retrospective observational design, all included patients had noncommunicating hydrocephalus, patients received ETV or VS, and the study reported at least one of the following outcomes: improvement of symptoms, major complications, hematoma, infection, reoperation, mortality, duration of surgery, and hospital stay. Additionally, the following exclusion criteria were employed and listed as follows: reviews and study reported repeated or overlapped publications.

### 2.2 Data collection and quality assessment

Two authors independently extracted the relevant information via a standardized data extraction form, including first author's name, publication year, country, study design, sample size, mean age, percentage male, mean Endoscopic Third Ventriculostomy Success Score, disease status, follow-up duration, and reported outcomes. The Newcastle–Ottawa scale (NOS), which is quite comprehensive and has been partially validated for assessing the quality of observational studies included in meta-analysis, was used to evaluate methodological quality.^[22]^ The NOS is based on selection (4 items), comparability (one item), and outcome (3 items). When there was disagreement between the 2 investigators on data eligibility and quality assessment, information was examined and adjudicated independently by an additional author referring to the original studies.

### 2.3 Statistical analysis

The summary odds ratios (ORs) and 95% confidence intervals (CIs) were employed in the pooled analysis for symptom improvement, major complications, hematoma, infection, reoperation, and mortality based on events occurring in each group using random-effects models.^[23,24]^ Further, the standard mean difference (SMD) with 95%CI was used to calculate the summary results for the duration of surgery and hospital stay using random-effects models.^[23,24]^ Heterogeneity between studies was investigated using the *I*^2^ and *Q* statistics, and we considered *I*^2^ value > 50% or *P* values < .05 as indicative of significant heterogeneity.^[25,26]^ Sensitivity analyses were conducted for symptom improvement and major complications to assess the influence of a single study in the meta-analysis by removing each individual study sequentially.^[27]^ Subgroup analyses were conducted for symptom improvement and major complications based on publication year, country, study design, sample size, mean age of patients, and study quality. Funnel plots, Egger et al,^[28]^ and Begg and Mazumdar tests^[29]^ were employed to qualitatively and quantitatively calculate potential publication bias. Two-tailed *P* < .05 was accepted as statistically significant. All analyses were conducted using STATA version 10.0 (Stata Corp LP, College Station, TX).

## 3 Results

### 3.1 Literature search

**Figure 1:**
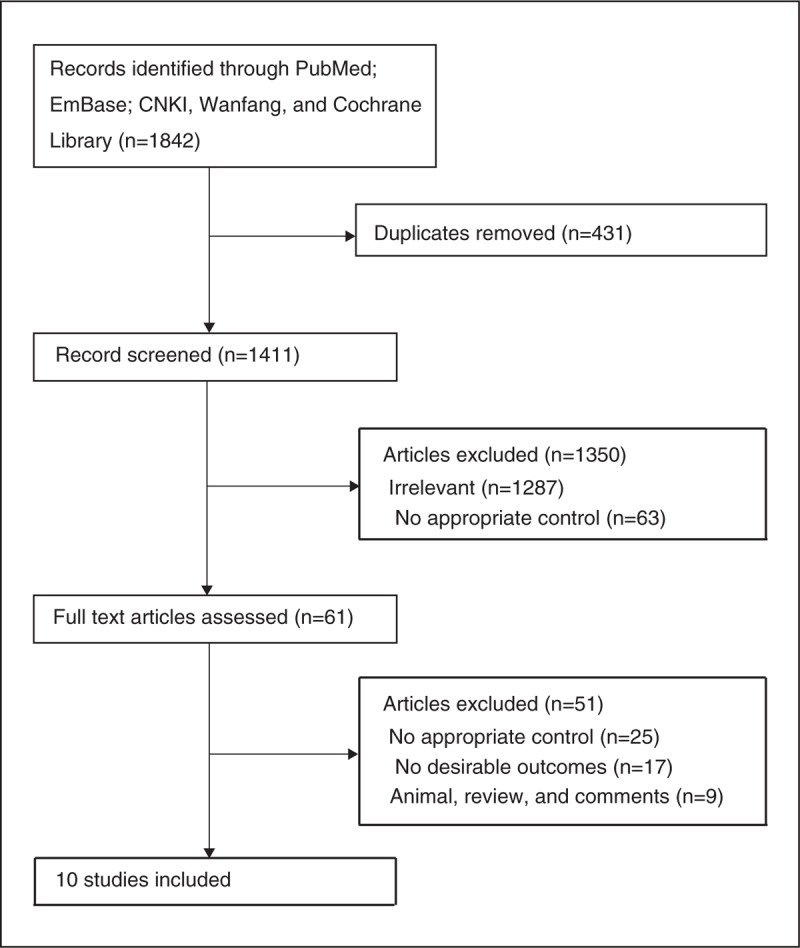
The flow diagram of screened, excluded, and analyzed publications.

The flow diagram of the study selection process is presented in Figure 1. A total of 1842 articles were retrieved from the initial literature search, of which 1,781 were excluded as they were duplicate or irrelevant. A total of 61 potentially eligible studies were selected. After excluding 51 studies (no appropriate control, no sufficient data, and animal studies, review, or comments), 10 observational studies comparing the efficacy and safety of ETV and VS for noncommunicating hydrocephalus were included in the final analysis.^[30–39]^ A manual search of the reference lists of these studies did not yield any new eligible studies. The characteristics of the included studies are presented in Table 1.

**Table 1:**
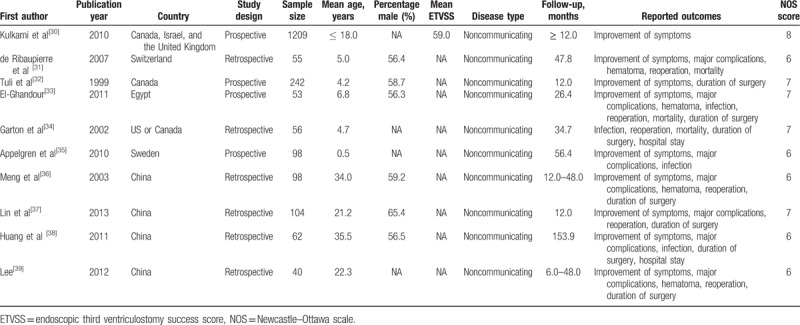
Baseline characteristic of studies included in the systematic review and meta-analysis.

### 3.2 Study characteristics

Of the 10 included studies (with a total of 2017 patients), 4 had a prospective design and the remaining 6 had a retrospective design. The studies were published between 1999 and 2013 and the sample sizes ranged from 40 to 1209. Five studies were conducted in developed countries, including Canada, Israel, the United Kingdom, Switzerland, the United States, and Sweden, and the remaining 5 studies were conducted in developing countries, including Egypt and China. Nine studies investigated the incidence of symptom improvement, 7 studies the incidence of major complications, 5 reported on hematoma, 5 reported on infection, 6 reported on reoperation, 3 reported on mortality, 7 reported on duration of surgery, and 2 reported on hospital stay. Study quality was assessed using the NOS score and the relevant results are presented in the last column of Table 1. Here, we considered a study with a score ≥ 7 as being of high quality. Overall, one study had a score of 8, 4 had a score of 7, and the remaining 5 had a score of 6.

### 3.3 Meta-analysis

**Figure 2:**
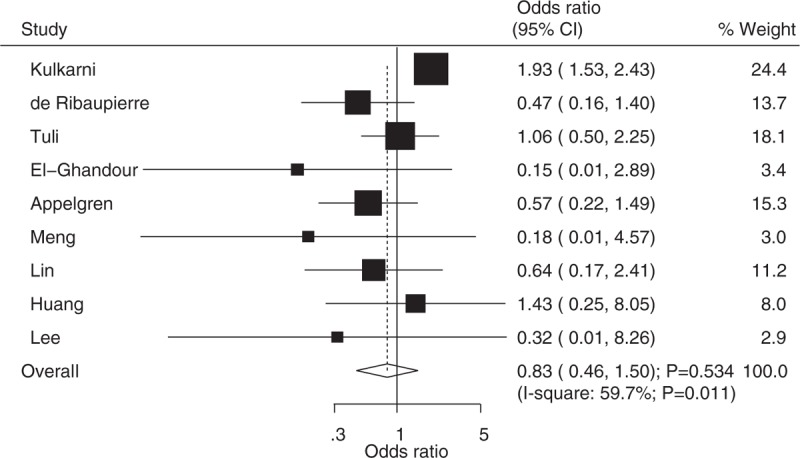
Effect of endoscopic third ventriculostomy on the incidence of improvement of symptoms compared with ventriculoperitoneal shunt.

**Figure 3:**
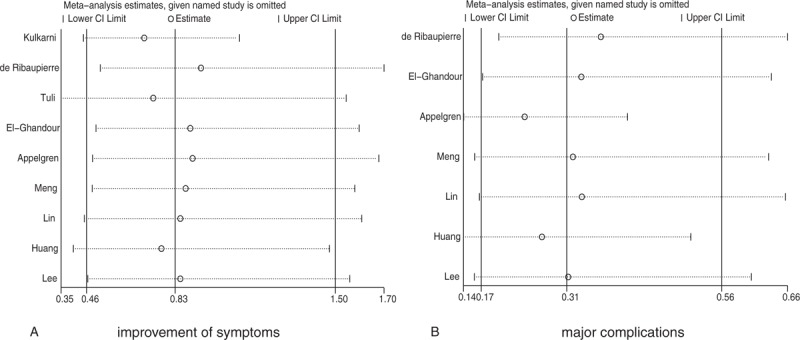
Sensitivity analyses for improvement of symptoms and major complications.

Data from nine studies were employed including 1169 events of symptom improvement and 1963 patients with noncommunicating hydrocephalus. We noted that ETV reduced the incidence of symptom improvement by 17%, but this reduction was not statistically significant (OR: 0.83; 95%CI: 0.46–1.50; *P* = .534; Fig. 2), and evidence of potentially significant heterogeneity was observed (*I*^2^ = 59.7%; *P* = .011). As a result, a sensitivity analysis was conducted and after each study was sequentially excluded from the pooled analysis, the conclusion was not affected by the exclusion of any specific study (Fig. 3).

**Figure 4:**
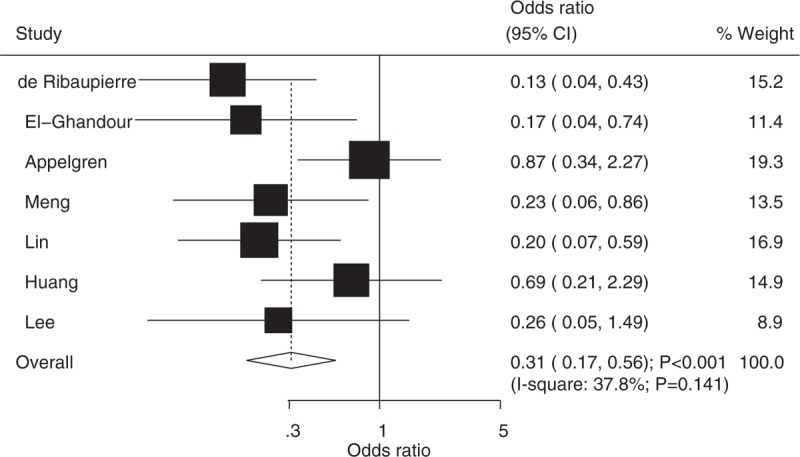
Effect of endoscopic third ventriculostomy on the incidence of major complications compared with ventriculoperitoneal shunt.

Data from 7 studies were employed including 161 events of major complications and 512 patients with noncommunicating hydrocephalus. We noted that ETV was associated with lower incidence of major complications when compared with VS (OR: 0.31; 95%CI: 0.17–0.56; *P* < .001; Fig. 4), and there was moderate heterogeneity across the included studies (*I*^2^ = 37.8%; *P* = .141). After sequential exclusion of each study from all pooled analyses, the conclusion was not affected by the exclusion of any specific study (Fig. 3).

**Figure 5:**
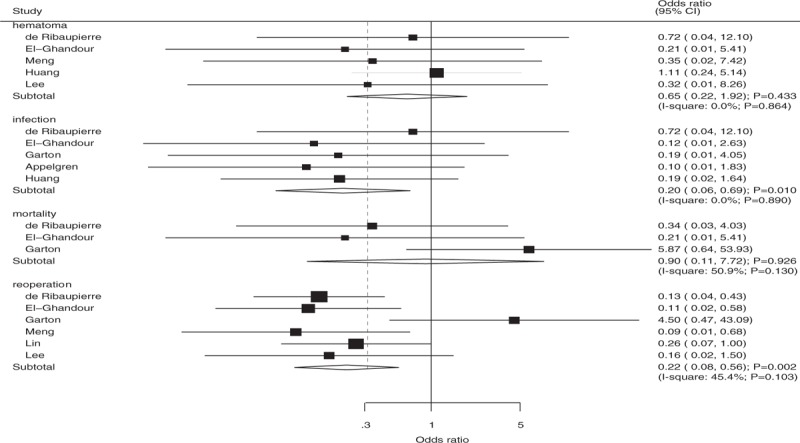
The summary results for hematoma, infection, reoperation, and mortality compared with ventriculoperitoneal shunt.

The number of studies available for each outcome was 5, 5, 6, and 3 studies for hematoma, infection, reoperation, and mortality, respectively (Fig. 5). Overall, there were no significant differences between ETV and VS for the outcomes of hematoma (OR: 0.65; 95%CI: 0.22–1.92; *P* = .433; [*I*^2^ = 0.0%; *P* = .864]) and mortality (OR: 0.90; 95%CI: 0.11–7.72; *P* = .926; [*I*^2^ = 50.9%; *P* = .130]). Further, ETV were associated with lower incidence of infection (OR: 0.20; 95%CI: 0.06–0.69; *P* = .010; [*I*^2^ = 0.0%; *P* = .890]) and reoperation (OR: 0.22; 95%CI: 0.08–0.56; *P* = .002; [*I*^2^ = 45.4%; *P* = .103]) when compared with VS.

**Figure 6:**
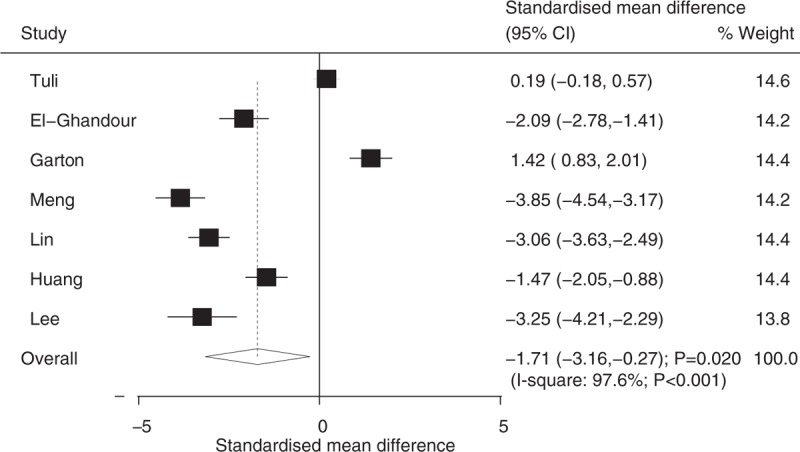
Effect of endoscopic third ventriculostomy on the duration of surgery compared with ventriculoperitoneal shunt.

**Figure 7:**
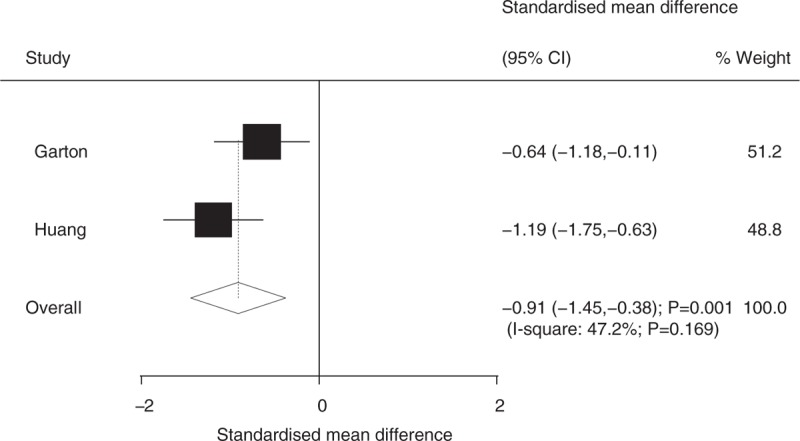
Effect of endoscopic third ventriculostomy on hospital stay compared with ventriculoperitoneal shunt.

Seven studies reported on the duration of surgery, and 2 studies reported on hospital stay. We noted that ETV was associated with shorter duration of surgery (SMD: −1.71; 95%CI: −3.16 to −0.27; *P* = .020; [*I*^2^ = 97.6%; *P* < .001]; Fig. 6) and hospital stay (SMD: -0.91; 95%CI: −1.45 to −0.38; *P* = .001; [*I*^2^ = 47.2%; *P* = .169]; Fig. 7) compared with VS.

### 3.4 Subgroup analyses

Subgroup analyses were conducted for symptom improvement and major complications to minimize heterogeneity among the included studies and to evaluate the treatment effects of ETV versus VS in patients with specific characteristics (Table 2). We noted that ETV was associated with lower incidence of symptom improvement when the sample size of study was less than 100 (OR: 0.53; 95%CI: 0.29–1.00; *P* = .049). Further, the results of the interaction tests suggested that publication year, country, study design, sample size, and study quality were significantly associated with the incidence of symptom improvement. Further, most subset test results suggested that ETV was associated with lower incidence of major complications except in studies conducted in developed countries, those with prospective designs, and those that included patients with a mean age < 18.0 years.

**Table 2:**
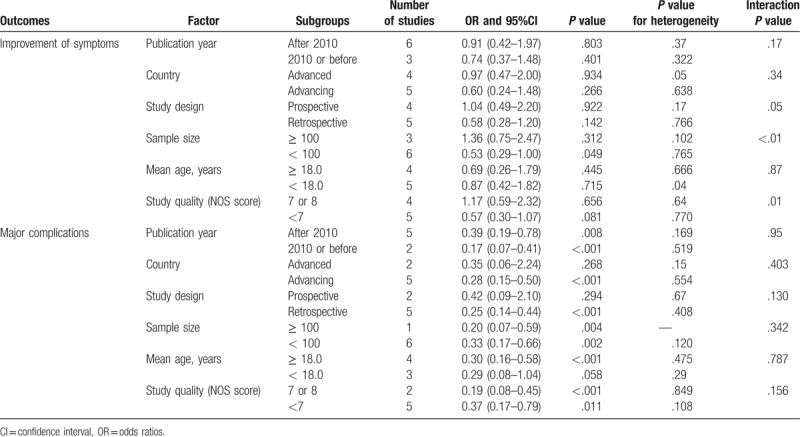
Subgroup analyses for improvement of symptoms and major complications.

### 3.5 Publication bias

**Figure 8:**
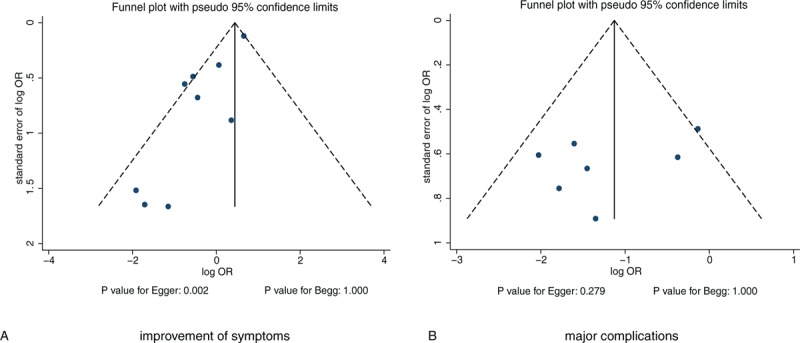
Funnel plots for improvement of symptoms and major complications.

Review of the funnel plots could not exclude the potential for publication bias for symptom improvement and major complications (Fig. 8). We noted no significant publication bias for major complications (*P* value for Egger: .279; *P* value for Begg: 1.000). Although the Begg test showed no evidence of publication bias for symptom improvement (*P* = 1.000), the Egger test showed potential evidence of publication bias (*P* = .002). The results did not change after adjustment using the trim-and-fill method, and the adjusted OR was 0.83 (95%CI: 0.46–1.50; *P* = .534).^[40]^

## 4 Discussion

Our current study was based on observational studies and comparisons of ETV and VS on the outcomes of symptom improvement, major complications, hematoma, infection, reoperation, mortality, duration of surgery, and hospital stay. This large quantitative study included 2,017 individuals from 4 prospective studies and 6 retrospective studies involving a broad range of populations. Findings from this meta-analysis suggest that ETV was not significantly associated with the incidence of symptom improvement, hematoma, and mortality, while it could reduce the risk of major complications, infection, and reoperation. Further, we noted that ETV was associated with shorter duration of surgery and hospital stay. Finally, subgroup analyses found that ETV was associated with lower risk of major complications in most subsets and that it had a protective role in symptom improvement when the sample size was < 100.

Except for the incidence of mortality, a previous meta-analysis of 8 studies provided similar results to those of the current study. However, they utilized the weight mean difference to summarize continuous data with substantial heterogeneity instead of using the SMD for the summary results. Finally, comparisons of treatment effects for ETV and VS in patients with specific characteristics were not conducted using stratified analysis.^[19]^ Another important meta-analysis only provided the incidence of failure rate and focused on qualitative analysis.^[20]^ Therefore, we conducted a comprehensive and quantitative analysis of available studies to compare the efficacy and safety of ETV and VS for patients with noncommunicating hydrocephalus.

There was no significant difference between ETV and VS for the incidence of symptom improvement. However, the study conducted by Kulkarni et al^[30]^ suggested that the risk of failure in ETV becomes progressively lower than that in VS and is associated with increased duration of surgery. Subgroup analysis suggested that there was a significant difference in symptom improvement when the study sample size was less than 100. The possible reason for this could be that the Kulkarni et al. study had a larger sample size and the number of included studies with sample sizes lower than 100 was large, which is associated with a higher power to detect differences between ETV and VS on symptom improvement.

In this study, we found that ETV was associated with lower incidence of major complications. Nearly all included studies reported similar results. The specific types including bleeding, memory disturbance, focal neurological deficits, third nerve palsy, hypothalamic dysfunction, and combined complication events were associated with higher statistical power, facilitating the acquisition of significant differences between ETV and VS. Subgroup analyses suggested that ETV was not superior to VS in terms of major complications in studies conducted in developed countries, with prospective designs, which included patients with mean age < 18 years. The possible reason for this could be the lower number of included studies in these subgroups, especially if event rates were lower than were expected in individual study, which always acquired no statistically significant result.

There was no significant difference between ETV and VS and the risk of hematoma and mortality. The reason for this could be that few events occurred in each group, and these studies designed symptoms indexes as primary outcomes, and the sample sizes of these studies were not sufficient large to detect significant differences for hematoma and mortality. Furthermore, we noted that ETV was correlated with lower incidence of infections and reoperation. The possible reason for this could be that VS was associated with higher failure rate due to infection and malfunction. Finally, ETV was associated with shorter duration of surgery and hospital stay when compared with VS, while it was not significantly less costly.

The limitations of our study are as follows: all reported outcomes were calculated with raw data, and the adjusted results were not available, which may play an important role on the treatment effects for patients with noncommunicating hydrocephalus; information on the specific causes of hydrocephalus was not available, and the treatment effects between ETV and VS according to specify causes were not calculated; the results of subgroup analyses in mostly subsets based on smaller number of studies, and these results may be unreliable. The current study was based on published studies, and publication bias is an inevitable issue; and the analysis used pooled data and individual data were not available, which precluded more detailed relevant analysis, which would have allowed us to obtain more comprehensive results.

In summary, the results of this meta-analysis suggested that ETV was not superior to VS in terms of symptom improvement. Further, ETV could reduce major complications in patients with noncommunicating hydrocephalus. Although there were no significant differences related to hematoma and mortality, ETV was associated with lower incidence of infection and reoperation. Finally, patients who received ETV had shorter duration of surgery and hospital stay. Future large-scale randomized controlled trials should be conducted focusing on specific adverse events and on the evaluation of treatment effects in patients with specific characteristics.

## Author contributions

**Conceived and designed the experiments**: Lin Jiang and Yanfeng Zhou.

**Performed the experiments**: Lin Jiang, Guangzhong Gao, and Yanfeng Zhou.

**Analyzed the data**: Lin Jiang.

**Contributed reagents/materials/analysis tools**: Yanfeng Zhou.

**Wrote the paper**: Lin Jiang and Yanfeng Zhou.

**Conceptualization:** Lin Jiang.

**Data curation:** Lin Jiang, Yanfeng Zhou.

**Formal analysis:** Lin Jiang, Guangzhong Gao, Yanfeng Zhou.

**Investigation:** Lin Jiang, Yanfeng Zhou.

**Methodology:** Lin Jiang, Yanfeng Zhou.

**Project administration:** Lin Jiang, Yanfeng Zhou.

**Resources:** Lin Jiang, Yanfeng Zhou.

**Software:** Lin Jiang, Guangzhong Gao.

**Supervision:** Lin Jiang, Guangzhong Gao.

**Validation:** Lin Jiang.

**Visualization:** Lin Jiang.

**Writing – original draft:** Lin Jiang.

**Writing – review & editing:** Lin Jiang.
